# *In Vivo* Study of the Effects of Peptide-Conjugated Near-Infrared Fluorescent Quantum Dots on the Tumorigenic and Lymphatic Metastatic Capacities of Squamous Cell Carcinoma Cell Line Tca8113 and U14

**DOI:** 10.3390/ijms11041413

**Published:** 2010-03-31

**Authors:** Zhi-Gang Li, Kai Yang, Yu-An Cao, Gang Zheng, De-Ping Sun, Cheng Zhao, Jia Yang

**Affiliations:** 1 Department of Oral and Maxillofacial Surgery, the First Affiliated Hospital, Chongqing Medical University, Chongqing 400016, China; 2 Chongqing Hospital of Traditional Chinese Medicine, Chongqing 400013, China

**Keywords:** peptide, near-infrared fluorescence, quantum dots, tumor, growth, proliferation, apoptosis, metastasis

## Abstract

Quantum dots (QDs) have great potential in non-invasive monitoring and imaging of tumor cells *in vivo*, but it is unknown if QDs affect their tumorigenesis and metastasis. Here, we applied peptide-conjugated near-infrared fluorescent QDs (NIRF-QDs) to label the squamous cell carcinoma cells Tca8113 and U14. We tested the proliferation and apoptotic capacities of both cells, and the capacity of cervical lymph node metastasis after tumorigenesis in U14 cells’. We find that QDs do not affect the tumor cells’ capacities to grow, proliferate, and metastasize. Our study provides critical data to support the application of NIRF-QDs in non-invasive monitoring and imaging of tumor cells *in vivo*.

## 1. Introduction

Real-time dynamic visual monitoring of the proliferation and metastasis of tumor cells *in vivo* through direct and non-invasive methods has become one of the key technologies in the studies of tumor formation and development, early diagnosis and treatment. Recently developed quantum dots (QDs) has shown great development potential in this field [1–3].

As a new type of nano-fluorescent material, QDs, has many unique optical properties compared to the traditional fluorescent markers, such as narrow excitation spectra but wide emission spectra, strong fluorescence, high photochemical stability, and resistance to photolysis or bleaching, due to the effects of quantum size and dielectric confinement [4–7]. These optical properties can be used to dynamically trace live cells for a prolonged time, while all fluorescent probes currently being used cannot (a variety of organic fluorescent dyes and fluorescent proteins) [8]. Especially the fluorescence of recently developed QDs with near-infrared light of emission wavelength (700–900 nm) not only can strongly penetrate human tissue, but also avoids the interference of autofluorescence from the tissue. This is particularly suitable for *in vivo* non-invasive medical imaging [9–11]. Currently, QDs are used to label tumor cells by non-specific endocytosis or by linking with antibodies, ligands, and peptides to form fluorescent probes for the *in vivo* imaging of non-invasive tumor cells [8,12–14], sentinel lymph node detection [15,16], and the targeting tumor angiogenesis [17]. These studies clearly demonstrated the great potential of the excellent optical characteristics of QDs in the fields of *in vivo* non-invasive *in situ* study of the formation and development of cancer cells, and the early diagnosis and treatment of cancer.

It has been shown that bio-labeling of tumor cells with QDs is not toxic and does not affect their growth and differentiation [1,8]. Other reports applied QDs to label tumor cells and monitored their invasion and metastasis by *in vivo* imaging [8,12–15]. All those studies demonstrated that QDs are a good agent for *in vivo* imaging and can be used to non-invasively track the growth and metastasis of tumor cells *in vivo*. However, one important question that remains unanswered is whether QDs affects tumor cell growth and metastasic capacities after labeling. This question is very important because it is directly related to the concern as to whether tumor cell imaging using QDs labeling can truly reflect the *in vivo* situation of tumor cell invasion and metastasis. No previous reports have addressed this critical issue.

In our previous report, we used peptide-conjugated Qtracker™ QD800 to successfully label human tongue squamous carcinoma cells (Tca8113 cells). Plate colony formation assay, transwell invasion chamber assay, and erosion experiment verified that labeling by QD800 does not affect the growth, proliferation, invasion, and metastasis of Tca8113 cells *in vitro* [18]. Based on those *in vitro* results, we used Qtracker™ QD800 to label the Tca8113 and highly metastatic cervical squamous carcinoma cells (U14 cells) of Kunming mouse through endocytosis. We then inoculated those labeled cells in mice and observed the changes of their tumorigenic capacities and lymphatic metastatic capacities *in vivo*. Our results provide critical information to support the application of QDs in non-invasive imaging of tumor cells.

## 2. Experimental Section

### 2.1. Materials

#### 2.1.1. Instruments and Reagents

Human tongue squamous cell carcinoma cell line Tca8113 was from West China College of Stomatology, Sichuan University. Kunming mouse lymphatic high metastatic cervical squamous carcinoma cell line U14 was from Chinese Academy of Medical Sciences Cancer Institute. Qtracker™ 800 Cell Labeling Kit was from Invitrogen, U.S.A. Laser scanning confocal microscope (TCS-SP5) was from Leica, Germany. Flow cytometry machine (FACSVantage) was from BD, U.S.A. Low-speed refrigerated centrifuge (Z233MK-2) was from HERMLE, Germany.

#### 2.1.2. Experimental Animals

Five female SPF level BALB/c nu/nu substrain nude mice, 5–6 weeks old and weighing 19–20 g, were purchased from the Experimental Animal Center of Chongqing Medical University. They were kept under conditions of constant temperature and humidity, and sterile bedding, food, and water. Fifteen female Kunming mice, 5–6 weeks old and weighing 20–21 g, were purchased from the Experimental Animal Center of Chongqing Medical University. They were kept at room temperature.

All experimental procedures were approved by the Laboratory Animal Management Committee of Experimental Animal Research Institute.

### 2.2. Experimental Methods

#### 2.2.1. QDs labeling of Tca8113 and U14 cells

The labeling of Tca8113 and U14 cells by QD800 was carried out following the manual of Qtracker™ 800 Cell Labeling Kit, which is briefly described as follows. Tca8113 and U14 cells were trypsinized and transferred to 6 EP tubes (1 × 10^6^ cells / tube) with 3 for each cell line. Then 0.2 mL freshly prepared 10 nM of QD800 labeling solution was added to each tube. After even mixing, the cells were incubated for 1 h and centrifuged for 5 min (1000 r/min at 4 °C). The medium was discarded and the pelleted cells were washed by PBS twice to remove the QDs that failed to enter the cells, and were plated on 6-well plates with medium for culturing. After 1 h, the cells were resuspended and detected for labeling efficiency (the average was used to calculate labeling efficiency); after 2 h, laser scanning confocal microscopy was used to observe the QD labeled Tca8113 and U14 cells.

#### 2.2.2. Tumorigenicity Experiment

After labeling, 0.1 mL of Tca8113/QD800 and Tca8113 cells (2 × 10^6^ cells / mL) was immediately inoculated subcutaneously on the left and right backs of 5 nude mice, respectively, and 0.1 mL of U14/QD800 and U14 cells (2 × 10^6^ cells / mL) was immediately inoculated subcutaneously on the left and right backs of 5 Kunming mice, respectively. Tumor formation was monitored. 24 days and 14 days after inoculation, nude mice and Kunming mice were sacrificed by cervical dislocation, and the tumors were taken out and weighed. The tumor’s maximal diameter (a) and minimal diameter (b) were measured, and the volume of the tumor was calculated by the equation of V = 0.5 × a × b^2^. Twenty tumors were saved for the following detections of proliferation and apoptosis.

#### 2.2.3. Detection of Cell Proliferation and Apoptotic Capabilities of Tumorigenic Cells *in Vivo*

Tumor cells from the 20 fresh tumor tissues (Tca8113/QD800, Tca8113, U14/QD800, and U14; 5 specimens each) were suspended, and their proliferation and apoptosis were analyzed by flow cytometry. A brief description of the experimental procedure is as follows: each sample was cleaned by removing the surrounding necrotic tissues, washed 3 times with D-Hanks solution, and treated with penicillin (5 × 10^5^ U/L) and streptomycin (100 mg/L) for 20 min. Then each tumor was cut into 1 mm^3^ pieces, which were subsequently treated with digestion solution (20 mg/10 mL of collagenase, 10 mg/10 mL of DNase, and 0.002 mg/10 mL of Hyaluronidase) for 40 min and filtered with 200-mesh tantalum net. The filtered solutions were centrifuged at 1,000 r/min for 5 min and the pellets were washed twice with PBS and then resuspended in RPMI 1,640 medium for flow cytometry analyses.

Detection of cell proliferation: all cell concentrations were adjusted to 1 × 10^6^/mL and 1 mL of cell suspension solution was centrifuged (1,000 r/min, 4 °C) for 5 min. The pelleted tumor cells were fixed in 70% ethanol at −20 °C and then kept at 4 °C overnight. The cells were spun down by centrifuging at 1000 r/min at 4 °C for 5 min, followed by 2 washes with PBS and 30 min staining with PI (Propidium iodide) solution at 4 °C in the dark. The following equation was used to calculate the proliferation index of tumor cells (PI): PI = (S + G2/M)/(G0/G1 + S + G2/M) × 100%.

Detection of cell apoptosis: all cell concentrations were adjusted to 1 x 10^6^/mL and 1 mL of cell suspension solution was centrifuged (1,000 r/min, 4 °C) for 5 min. The pelleted tumor cells were washed twicewith 4 °C PBS, stained in 200 μL of Annexin V staining solution at room temperature for 15 min then in 1 mL of PI staining solution for 5 min before flow cytometry analysis. The following equation was used to calculate the apoptosis index of tumor cells (AI):

AI=(number of apoptotic cells/total number of measured cells) × 100%

#### 2.2.4. Analyses of the Effects of QDs on U14 CELLS’ Lymphatic Metastatic Capability

The method described above was followed to prepare U14/QD800 cells, which were immediately used for the following experiments. Ten Kunming mice were randomly and evenly divided into an experimental group and a control group, and 0.1 mL of U14/QD800 or U14 cells (both at 2 × 10^6^/mL) were injected to the mice from experimental and control groups, respectively, under the bilateral buccal mucosa to establish the model of Buccal cancer neck lymphatic metastasis. Twenty-two days later, the mice were sacrificed by cervical dislocation and all of their bilateral cervical lymph nodes were collected, counted, before being paraffin-embedded, sliced, stained with HE, and analyzed for cervical lymph node metastasis.

## 3. Results

### 3.1. QDs Labeling of Tca8113 and U14 Cells

**Figure 1.**
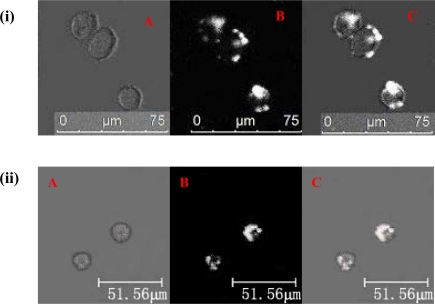
Fluorescent images of Tca8113 and U14 cells 2 h post labeling with QD800. Top panel: Tca8113 cells labeled with QD800; bottom panel: U14 cells labeled with QD800. (**A**) Bright-field, (**B**) QD800 fluorescence image, (**C**) overlay image.

The labeling rates of Tca8113 cells and U14 cells after one hour were 97.30 ± 1.12% and 97.72 ± 1.04%, respectively, as detected by flow cytometry. The average fluorescence intensities were 228.67 ± 7.36 and 229.96 ± 6.43 for Tca8113 and U14, respectively. Two hours after labeling, the laser scanning confocal microscopy showed that there were a large number of QD800 in both Tca8113 and U14 cells (Figure 1).

### 3.2. Tumorigenicity Experiment Demonstrates That Labeling Tca8113 and U14 Cells with QD800 Does Not Change the Tumorigenic Capacity

**Figure 2.**
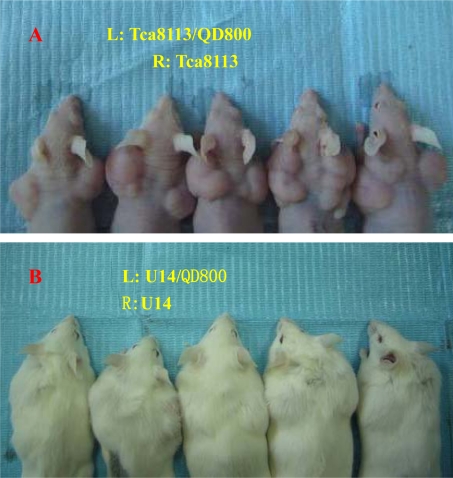
Tumorigenesis of QD800-labeled Tca8113 and U14 cells: (**A**) Tca8113/QD800 and Tca8113 cells ata concentration of 2 × 10^6^/mL were inoculated subcutaneously on the bilateral back of five nude mice and tumor growth was monitored 24 days later; (**B**) U14/QD800 and U14 cells at a concentration of 2 × 10^6^/mL cwere inoculated subcutaneously on the bilateral back of five Kunming mice and the tumor growth was monitored 14 days later.

Obvious tumor formation was observed in both the Tca8113/QD800 group and Tca8113 group 24 days after the subcutaneous inoculation, and both the U14/QD800 group and U14 group formed tumors 14 days after the inoculation (Figure 2). The average weights of tumors of the Tca8113/QD800 group was 1.31 ± 0.16 g and of the Tca8113 group was 1.35 ± 0.15 g, which are not significantly different from each other (*P* = 0.6941). The average sizes of tumors were 2.74 ± 0.66 cm^3^ and 2.54 ± 0.68 cm^3^ for the Tca8113/QD800 group and Tca8113 group, respectively, which are also not significant different from each other (*P* = 0.6496). The average weights of tumors of the U14/QD800 group and U14 group were 1.12 ± 0.06 g and 1.15 ± 0.12 g, respectively, which are not significantly different from each other (*P* = 0.6305). The average sizes of tumors were 2.30 ± 0.61 cm^3^ and 2.32 ± 0.46 cm^3^ for the U14/QD800 group and U14 group, respectively, which are also not significant different from each other (*P* = 0.9548). These results indicate that labeling of Tca8113 and U14 cells with QD800 does not change the tumorigenic capacity.

### 3.3. Detection of Proliferation and Apoptosis Indices of Tumorigenic Cells *in Vivo*

Twenty four days after tumorigenesis, the average proliferation indices of Tca8113/QD800 and Tca8113 cells were 46.21 ± 1.36% and 47.07 ± 1.44%, respectively, which are not significantly different from each other (*P* = 0.3600). Their average apoptosis indices were 13.54 ± 0.79% and 12.34 ± 0.68%, respectively, which are also not significantly different from each other (*P* = 0.3290). Fourteen days after tumorigenesis, the average proliferation indices of U14/QD800 and U14 cells were 61.53 ± 2.37% and 60.87 ± 2.13%, which are not significantly different from each other (*P* = 0.6556), and their average apoptosis indices were 14.19 ± 0.92% and 13.40 ± 0.76%, respectively, which are also not significantly different from each other (*P* = 0.1771). These results indicate that labeling of Tca8113 and U14 cells with QD800 does not change the proliferation and apoptosis of Tca8113 and U14 cells after tumorigenesis.

### 3.4. Effects of QDs on U14 Cells’ Lymphatic Metastatic Capacity

**Figure 3.**
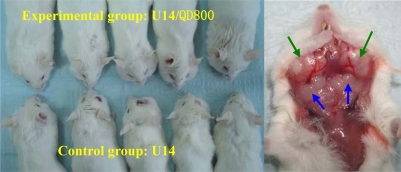
Buccal cancer cervical lymph node metastasis model. Green arrows point to buccal tumors and blue arrows point to enlarged lymph nodes.

**Figure 4.**
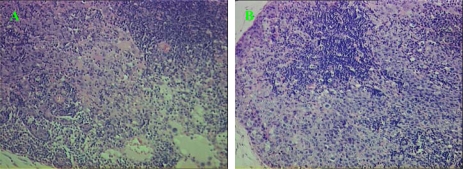
Histology of Buccal cancer cervical lymph node metastasis (HE staining, 200×). (**A**) U14/QD800 buccal cancer cervical lymph node metastasis; (**B**) U14 buccal cancer cervical lymph node metastasis.

Tumor formation was clearly observed in the U14/QD800 group and U14 group 22 days after inoculation in the cheek, and the bilateral neck regions both had significantly enlarged lymph nodes (Figure 3). 28 and 31 lymph nodes were found in the U14/QD800 group and U14 group, respectively, and 12 and 14 of them, respectively, had metastasis, (Figure 4). The lymph node metastasis rates were 42.86% and 45.16% for the U14/QD800 group and U14 group, respectively, which are not significant different (*P* = 0.9326). This indicates that labeling of U14 cells with QD800 does not change the lymphatic metastatic capacity of U14 cells.

## 4. Discussion

QDs have great potential in long term non-invasive monitoring and imaging *in vivo* because of their high light stability and high fluorescence quantum yield, compared to traditional organic fluorescent dyes and fluorescent proteins [1–3]. In recent years, many studies have demonstrated that the QDs with cell-penetrating peptides is suitable for various types of cell markers. It not only can quickly and efficiently label the cells, but also imposes no damage to living cells [19–22]. In this study, Qtracker™ QD800 were made of CdTe cores with Zns coating. Their surface was conjugated with a polycationic peptide(arginine 9-mer) to improve the cell uptake efficiency. We co-cultured Qtracker™ QD800 with Tca8113 and U14 cells. Our results show that peptide-conjugated QDs could rapidly enter and label the cells, and one hour after the labeling, 97.30% of Tca8113 cells and 97.72% of U14 cells were successfully labeled. Near-infrared fluorescent QDs have been shown to be a good choice for non-invasive imaging to monitor the growth and metastasis of tumor cells *in vivo* [13–17], and our previous report found that QDs does not affect the invasion and metastasis of cancer cells *in vitro* [18]. However, the effects of QDs on the tumorigenic and metastatic capacities of tumor cells *in vivo* requires investigation, because it directly determines whether the results obtained using this method truly reflect what is really happening for the tumor cells. In this study, we used Qtracker™ QD800 to label the human tongue Squamous Cell Carcinoma Cell Line Tca8113 and the Kunming mouse lymph node highly metastatic Squamous Cell Carcinoma Cell Line U14. After the labeling, we subcutaneously inoculated Tca8113/QD800 cells in nude mice with immune deficiency, and inoculated U14/QD800 cells in Kunming mice with normal immunity subcutaneously and under buccal mucosa with extensive lymphatic tubes. Our results indicate that QDs does not significant affect the tumorigenic capacities of Tca8113 and U14 cells and the lymphatic metastatic capacity of U14 cells *in vivo*. We also found that labeling of Tca8113 and U14 cells with QD800 did not change the proliferation and apoptosis of Tca8113 and U14 cells *in vivo*. Our study provides scientific basis for the application of QDs in the imaging study of tumor formation, development, movement, and metastasis.

## 5. Conclusion

QDs have shown great potential in non-invasive monitoring and imaging of tumor cells *in vivo* [1–3]. However, the question of whether QDs affect the tumorigenic and metastatic capacities of tumor cells becomes critical in application of QDs labeling in non-invasive imaging studies. We have demonstrated *in vitro* that QDs do not change tumor cells’ invasion and metastasis, however, this requires confirmation in *in vivo* studies. We applied peptide-conjugated near-infrared fluorescent QDs to label Tca8113 and U14 tumor cells. We performed *in vivo* tumor formation assays, cervical lymph node metastasis assays, and other related experiments, and found that QDs labeling did not affect the growth, proliferation, and metastasis of those tumor cells. Our study provides critical data to support the application of near-infrared fluorescent QDs in non-invasive imaging and monitoring of tumor cells *in vivo*.
